# Measles Immunization Policies and Vaccination Coverage in EU/EEA Countries over the Last Decade

**DOI:** 10.3390/vaccines8010086

**Published:** 2020-02-14

**Authors:** Marco Montalti, Anna Kawalec, Erica Leoni, Laura Dallolio

**Affiliations:** 1 Department of Biomedical and Neuromotor Sciences, Unit of Hygiene, Public Health and Medical Statistics, University of Bologna, via San Giacomo 12, Bologna 40126, Italy; marco.montalti7@studio.unibo.it (M.M.); erica.leoni@unibo.it (E.L.); 2 Department of Hygiene, Wroclaw Medical University, Mikulicza-Radeckiego 7, Wroclaw 50-345, Poland; anna.kawalec@student.umed.wroc.pl

**Keywords:** measles, vaccination coverage, mandatory vaccination, recommended vaccination, European countries

## Abstract

Background: Starting from 2016, a major measles epidemic affected EU/EEA countries, after the measles incidence rate had progressively decreased from 2011 to 2015. Methods: This study describes measles incidences (ECDC reports), the vaccination coverages (VCs) (WHO/UNICEF reports) and the vaccination strategies, whether mandatory or recommended (ECDC Vaccine Scheduler), in 30 European countries over the last decade. Results: VCs were higher in countries with historically mandatory vaccination. However, in these countries, VCs declined between 2010 and 2018, in two cases to levels below 90% at the second dose. Instead, 9 and 12 countries with recommended vaccination increased their VCs, respectively, for the first and the second dose. Overall, the countries with VC ≥ 95% decreased from 20 to 15 for the first dose and from 10 to 7 for the second dose. This trend led Italy, France and Germany to make vaccination mandatory. In Italy this provision was introduced in 2017, and together with the catch-up campaigns on children between 1 and 15 years at school entry, led immediately to a strong effect: the first dose VC passed from 87% in 2016 to 93% in 2018, and from 82% to 89% for the second dose. Conclusions: Mandatory vaccination is certainly a policy producing positive effects; however, it seems to require additional strategies in order to reach the WHO goal of 95% of VC. Measures such as catch-up action on susceptible populations and communication strategies aimed at increasing awareness and acceptance should be considered.

## 1. Introduction

The 2012 to 2020 Global Measles and Rubella Strategic Plan was developed in order to achieve measles elimination in five WHO regions by 2020 [1]. Nonetheless, at present, the World Health Assembly has not endorsed a global measles goal and measles outbreaks continue to spread rapidly around the world [2].

In the last four years, a major measles epidemic affected the European Union (EU) member states and the European Economic Area (EU/EEA), recording 51,556 cases between 1 January 2016 and 30 September 2019. When compared with the 26,120 cases from the previous four years (2012–2016), these changes show an increase that could be interpreted as an alarming problem. Since October 2016, a measles resurgence has been observed in the EU/EEA, with outbreaks in several countries reported to the European Centre for Disease Prevention and Control (ECDC) and described in the literature [3,4,5,6]. In addition, many countries outside the EU/EEA are still experiencing significant and/or unforeseen outbreaks of measles. According to the latest data based on monthly reports from the World Health Organization (WHO), the highest number of measles cases reported annually since 2006 was achieved globally in the first six months of 2019 [7].

Based on the latest ECDC reports, the risk of measles spreading in the EU/EEA exists and is still high due to a significant number of non-immunized people. Among the main causes, there is a low vaccination coverage (VC) to which the emerging phenomenon of vaccine hesitancy has contributed in recent years [8]. According to the ECDC estimates, if we consider children and young people born in the 30 EU/EEA countries since 1999, 4.4% of the total (over four and a half million individuals) would not be immune to measles [9]. However, the number of susceptible people in the EU/EEA is much higher: A significant burden of measles is in fact affecting the neonatal and adult population. It is therefore necessary to consider both infants, too young for vaccination and without maternal antibodies, and adults who have never received the vaccine.

While average annual notification rates for newborns were up to 44 times higher than any other age group between 2016 and 2019, it is adults who are the most affected population group in 19 out of 30 European countries. There has also been a progressive increase in the median age over the last 10 years: From 10 years in 2009 to 17 years in 2019 [8]. The resulting increase of the susceptible population may be due to a build-up of susceptible adults as well as a rise in population movements. In fact, among the main risk factors for the spread of the infection at European level, it is worth mentioning the ongoing potential of imports and migrations, which may not only make it more difficult to manage existing outbreaks, but could also start new ones in countries with pockets of the non-immune population. In the three-year period 2016–2019, more than 40% of measles cases imported into EU/EEA countries came from another EU/EEA country, mainly from those affected by extensive measles outbreaks [9].

The goal of this study is to describe the incidence of measles, vaccination coverages and vaccination strategies in different European countries over the last decade. Starting from non-original data, but using data published by relevant international organizations as a source, the aim is to outline the progress of measles incidence and vaccination coverages in European countries that have been distinguished according to the vaccination policy adopted, whether mandatory or recommended.

## 2. Materials and Methods

A complete picture of new measles cases is constantly available, as the recorded cases are transmitted to the supranational surveillance institutions on a monthly basis from 30 EU/EEA countries. Data for new cases per million inhabitants were therefore downloaded from the ECDC monthly and annual reports (https://www.ecdc.europa.eu/en/publications-data), while information on current national immunization programmes were obtained from the “Vaccine Scheduler” application provided by the ECDC [10], adopting a definition of “mandatory” and “recommended” in accordance with the definition provided by Haverkate et al. in 2012 [11]. Data on the VC were downloaded from the WHO/UNICEF reports [12].

The study was conducted for new measles cases from 2011 (the first year in which all European countries except Croatia reported their data) to 2018 and for the VCs for the years 2010, 2016 and 2018. Finally, data were distinguished in relation to the vaccination strategies adopted in different countries during the same period.

## 3. Results

### 3.1. Incidence of Measles in Europe

In 2011, the incidence rate was 60/1,000,000, with a very uneven situation: From 0 cases recorded in Iceland and Cyprus and 0.4/1,000,000 in Latvia and Slovakia, to 187/1,000,000 in Romania and 234/1,000,000 in France. In the following years, cases gradually decreased, reaching incidence rates of around 7/1,000,000 in 2014 and 2015, before registering a new upward trend in 2016 (Table 1).

In 2017, the number of measles cases per million population increased to 28.3/1,000,000 (14,600 total cases) and continued to be characterised by great variability within the European scenario, with the highest incidence rates reported by Romania (283.8) and Greece (89.7), followed by Italy (84.0), Belgium (32.5) and Bulgaria (23.2). Some countries reported no new cases, such as Latvia and Malta, or a small number, such as Denmark, Estonia, Lithuania, Netherlands, Norway (<1/1,000,000).

Between 1 January 2018 and 31 December 2018, 30 EU/EEA member states reported 23.9 new cases of measles per million (12,352 total cases), 70% of which were laboratory confirmed. As many as 22 countries out of the 30 analysed recorded an increase in new cases per million inhabitants compared to 2017, with very remarkable variations for Greece (from 89.7 to 212.9 per million), France (from 7.8 to 43.5) and Slovakia (from 1.1 to 105.2) (Table 1).

**Figure 1:**
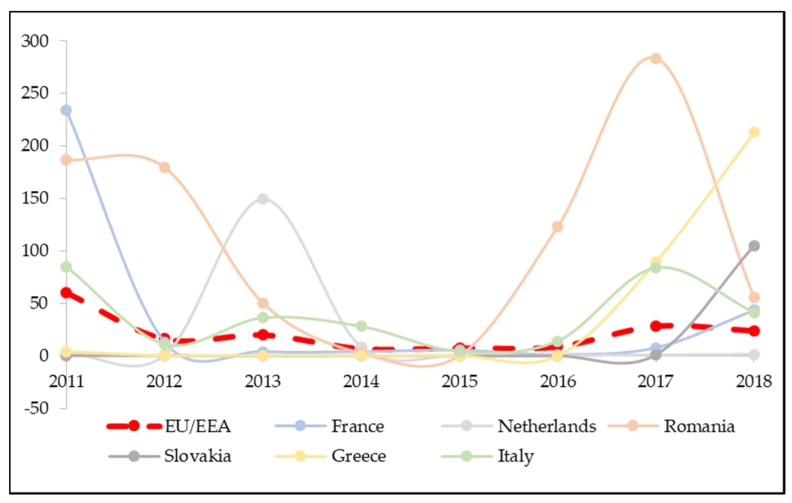
Trends of measles incidence rates between 2011 and 2018. Only the 6 countries reaching the highest annual incidence rates during the period under study were taken into account. The dashed-red curve represents the overall EU/EEA trend.

Three quarters (77%) of the total cases reported in EU/EEA in 2018 come from the five countries with the highest measles incidence rates. Among them are Greece (212.9) and Slovakia (105.2), much above the EU/EEA average (23.9), followed by Romania (55.3), France (43.5) and Italy (41.5). While the period under consideration might be too limited to monitor the characteristic peaks in the measles trend (every 3–4 years), for some countries, this trend can still be observed (Figure 1). France, Romania and Italy recorded peaks of incidence in 2011 with, respectively, 234, 187 and 85 measles cases per million inhabitants. Romania (283.8) and Italy (84.0) recorded a subsequent peak in 2017, while France recorded a peak in 2018 (Figure 1). In the last three years, a sharp increase can also be observed for Greece, from 0 cases in 2016 to 212.9 in 2018, and Slovakia (from 0 to 105.2). The incidence rates for Czech Republic (from 0.7 to 19.1), Cyprus (from 0 to 17.6) and Portugal (from 0 to 16.6) also show an evident increase in the last three years (Table 1).

If in 2011 there were 14 out of 29 countries with an incidence rate of less than 5 cases per million (one of the milestones to achieve measles elimination) in 2018, these fell to only 9 out of 30.

### 3.2. Measles Vaccination Coverage in Europe

**Figure 2:**
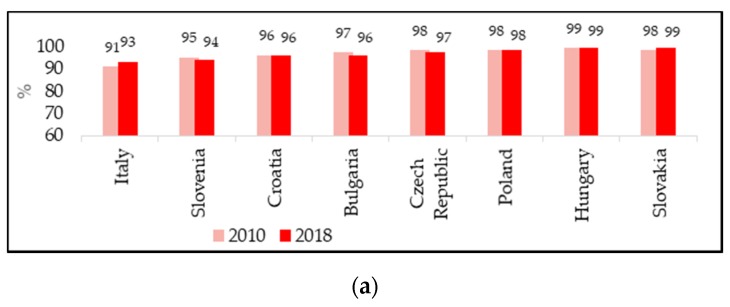

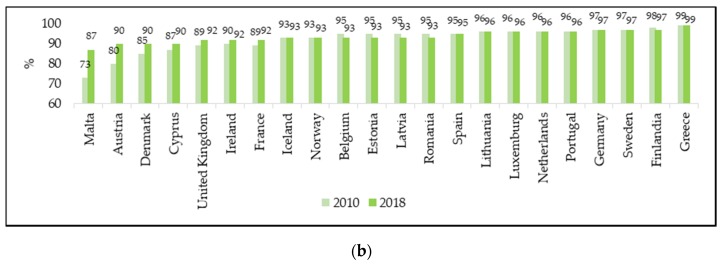
Vaccination coverage for the first dose of measles-containing vaccine in 2010 and 2018: (**a**) Countries with mandatory vaccination. Italy is listed among the countries with mandatory vaccination due to its change in vaccination policies in 2017; (**b**) countries with recommended vaccination (WHO/UNICEF reports). France is included in the recommended vaccination countries because vaccination, made mandatory for children born from 2018, has not yet been performed in the 2018 cohort.

**Figure 3:**
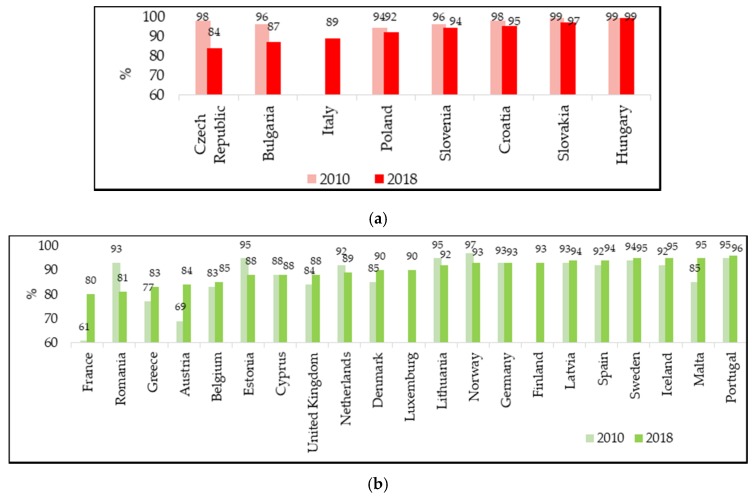
Vaccination coverage for the second dose of measles-containing vaccine in 2010 and 2018: (**a**) Countries with mandatory vaccination.Italy is listed among the countries with mandatory vaccination due to its recent change in vaccination policies in 2017. Vaccination coverage for the second dose of measles-containing vaccine in 2010 for Italy is not available; (**b**) countries with recommended vaccination (WHO/UNICEF reports). France is included in the recommended vaccination countries because vaccination, made mandatory for children born from 2018, has not yet been performed in the 2018 cohort.

The measles VCs for the first and the second dose were analysed in a total of 30 European countries, which were distinguished according to the vaccination policy adopted, whether mandatory or recommended. According to the objectives of the WHO 2012–2020 plan, VC with two doses of measles-containing vaccine should reach at least 95% to prevent the spread of the disease and to maintain an effective herd immunity [1]. This level of coverage was achieved for the first dose of measles-containing vaccine in 20 European countries in 2010 (Figure 2), while for the second dose only in 10 countries in the same year (Figure 3). Already in 2016, data showed a clear deterioration in the achievement of coverage, with 12 countries having adequate VC for the first dose and seven for the second. At the end of 2018, there was a very slight improvement compared to 2016, but was compared to 2010 for the first dose, with 15 countries having a VC of at least 95% (Figure 2), while only seven countries reached the target for the second dose (Figure 3).

It is important to mention that the recent European incidence rates can only be partly related to the lack of vaccination coverage in childhood, since WHO VC data are referred to children and recent measles outbreaks in Europe have mainly affected later ages (most recent median age is 17 years) [8]. However, it is not irrilevant that the countries that reported the highest annual incidence rates in at least one year in the period 2016–2018 (Table 1: Romania, Greece, Italy, France, Belgium Bulgaria, Czech Republic) had very low coverage rates at the second dose of vaccine (Figure 3a if mandatory: Bulgaria, Czech Republic, Italy) (Figure 3b if recommended: Belgium, France, Greece, Romania). The exception is Slovakia which, despite high VC rates for the second dose of vaccine, almost unchanged over the years (2010: 99%, 2017: 97%; 2018: 97%), recorded a sharp increase in new cases per million: From 1.1 in 2017 to 105.2 in 2018. This major variation could be explained by the fact that VC rates in the country are not uniform, and although generally at a good level, areas with coverage values well below 90% remain [13].

### 3.3. Comparison between Countries with Different Vaccination Policies

From 2010 to 2016, vaccination policies did not change in any European country. The seven countries where measles vaccination was mandatory belonged to the former Eastern Bloc (Bulgaria, Croatia, the Czech Republic, Hungary, Poland, Slovakia and Slovenia), characterized by the Soviet health system Semashko or its amendments [14]. Subsequently, the measles vaccine was made mandatory in Italy in 2017 and in France in 2018, and will be mandatory in Germany from 2020.

Comparing the VCs according to the vaccination policies adopted by different countries, it can be seen that, in 2010, the VCs for the first dose ranged from 95% to 99% if mandatory (all countries ≥ 95%) and from 73% to 99% if recommended (13 countries out of 22 ≥ 95%) (Figure 2). For the second dose, again in 2010, countries with mandatory vaccination had a coverage of 94% to 99% (6 countries of 7 > 95%), while countries with recommended vaccination from 61% to 97% (4 countries out of 21 ≥ 95%) (Figure 3). In the following years, VCs for the second dose gradually decreased in countries where vaccination was historically mandatory, although coverage levels remained, on average high, and higher than in countries where vaccination was recommended.

Table 2 shows the variations in VC at the second dose of vaccine in the last decade. Six out of seven countries with historically mandatory vaccination reduced their VCs from 2010 to 2016, and four of them also confirmed this trend from 2016 to 2018, in particular the Czech Republic and Bulgaria, which registered the highest decreases in the period 2010–2018. In the same overall period, 12 out of 19 countries with recommended vaccination increased their VCs. Very high coverage increases were observed already in 2016 in countries starting from very low VCs in 2010, such as Austria and France (Table 2).

Some countries, such as Italy and France, changed their immunization strategies by introducing some mandatory vaccinations, including measles. In Italy the measles vaccine became mandatory in 2017 along with 9 other vaccines. This measure had a strong impact on the VC, especially because of the new requirement for children and adolescents up to 16 years of age to have started the vaccination series in order to attend educational services. The 24 month measles VC (for children born in 2016) for the first dose reached 93.2% nationwide in 2018, increasing by 6.0% compared to 2016, although with some regional variations: 9 out of 20 regions exceeded 94% and only one had a VC below 90% [15]. The VC for the second dose, on the other hand, increased by 2.9% from June to October 2017 and then continued to grow until it reached 89.0% in 2018. At the same time, in Italy, there was a clear decrease in new cases, recording 41.5 cases per million in 2018 compared to 84.0 cases per million inhabitants in the previous year.

In France, home to one of the highest vaccine hesitancy rates in Europe (61% VC for the second dose of measle-containing vaccine in 2010), measles vaccination was made compulsory for newborns in 2018. This measure, being very recent and concerning only those born in 2018, cannot yet be analysed in terms of its impact on vaccination coverage.

## 4. Discussion

The analysis of the incidence rates of measles in Europe from 2011 to 2018 seems to indicate that the outbreak which occurred after 2016 reached a peak of cases in 2017; in 2018, although the overall incidence fell slightly, 22 countries experienced rates higher than those reported in 2017.

VCs were varied, but in general, they were higher in countries where vaccination was historically compulsory. There was a tendency in these countries to slightly reduce the VC rate over time, while countries with recommended vaccination, probably also as a result of the measures implemented for the increase in cases, in recent years recorded an increase in VC. Some countries achieved the VC recommended by the WHO plan 2012–2020 [1], even without having adopted the mandatory regime (in 2018: Sweden, Iceland, Malta, Portugal). This different trend in countries with different vaccination strategies confirms that adherence to vaccinations is affected by many other factors that have been highlighted in a recent report on measles surveillance worldwide—not only vaccine hesitancy, misinformation and lack of awareness of the need for vaccination, but political instability, limited availability of vaccines and financial barriers to receiving vaccination should also be considered [16].

The epidemic emergency, together with the decrease in the VCs, led some countries to change their policies. Italy was the first to move from a recommendation to a mandatory status, followed in 2018 by France and in 2020 by Germany. In 2017, the Italian Government approved the Law no. 119 that made ten vaccinations mandatory for children and adolescents up to 16 years of age [17]. The measure, adopted as part of the national response to a large measles outbreak and in order to counteract the decline in VCs, led to a significant increase in VC. This result was achieved, not only by making vaccination mandatory, but above all by strengthening vaccination services and implementing catch-up campaigns on children between 1 and 15 years of age at the time of enrolment in school [18]. The National Vaccine Prevention Plan (PNPV 2017–2019) and the regional immunization plans should guarantee a free service, not only to children, but also to target population groups, involving all stakeholders in catch-up activities: from public immunization services, to general practitioners and paediatricians engaged in local primary care services [19,20].

The policy of making vaccinations mandatory is very controversial [21,22]. Even in Italy this choice caused a wide public debate with conflicting positions, but contributed to increasing attention and confidence in vaccination thanks to the dissemination of information by qualified bodies, which counteracted the spread of misinformation that characterized the earlier years [23,24].

This study has some limitations. First of all, the presented data on incidence rates are affected by a possible underestimation, because the cases reported to the European Surveillance System (TESSy) may be incomplete. In particular, this may be the case for Romania, where the large epidemic caused delays in reporting [25]. Furthermore, the study is limited to a descriptive analysis of already published data from which it is not possible to derive correlations between vaccination strategies and vaccination coverage, and even less, between vaccination coverage and measles incidence rates. In fact, it should be noted that the VC coverage data referred to children and most of the cases in recent outbreaks involved adults. In addition, the period for which data are available is too short to clearly show the epidemic peaks every 3–4 years of typical of measles.

Despite these limitations, the data analysis allows some indications to be drawn. The mandatory nature of the vaccine is certainly a policy producing positive effects; however, it seems to require additional strategies in order to guarantee the goal stated by the WHO (VC at second dose ≥ 95%): Some countries where measles-containing vaccine is only recommended, such as Sweden, Iceland, Malta and Portugal, reach this goal, while countries with mandatory vaccination, such as the Czech Republic and Bulgaria have reduced their VCs to levels below 90%. This suggests that the introduction of mandatory vaccination should be accompanied by other actions aimed at ensuring a high-quality routine immunization programme, including an effective distribution of the vaccine, reminders, school campaigns, and all catch-up actions of the susceptible population. It would also be important to increase the control of the vaccination schedule and also offer immunization to older people, especially in professions that involve frequent direct contact with other people, such as school, health and social-health professionals.

## 5. Conclusions

EU/EEA countries experienced a decline in measles cases from 2011 to 2016. However, there was a resurgence of cases in recent years, which has necessitated the adoption of new prevention strategies. The introduction of mandatory vaccination is certainly a policy producing positive effects, as demonstrated by the case of Italy where mandatory vaccination has been accompanied by other fundamental measures such as the strengthening of vaccination services and the catch-up action on susceptible population groups. Communication strategies and measures such as health literacy promotion and empowerment are also fundamental tools to counteract vaccine hesitancy and to spread the culture of the individual value and social dimension of immunization.

## Figures and Tables

**Table 1:** New measles cases registered per million per country 2011–2018 (ECDC reports).

Country	Measles Cases per Million							
	2011	2012	2013	2014	2015	2016	2017	2018
Austria	12	2.3	8.9	13.3	35.2	3.1	10.9	8.8
Belgium	51	3.9	3.4	6.3	4.2	7.1	32.5	10.6
Bulgaria	21	0.1	2.2	0	0	0.1	23.2	1.8
Croatia	n.a.	n.a.	0.2	3.3	51.6	1.0	1.7	5.5
Cyprus	0	1.2	0	11.6	0	0	3.5	17.6
Czech Republic	2	2.1	1.3	21.1	0.9	0.7	13.8	19.1
Denmark	15	0.4	3.0	5.2	1.6	0.5	0.7	1.4
Estonia	5	3.0	1.5	0	3.0	1.5	0.8	7.6
Finland	5	0.9	0.4	0.6	0.2	0.7	1.8	2.7
France	234	13.2	4.2	4.1	5.5	1.2	7.8	43.5
Germany	20	2.0	21.7	5.4	30.5	4.0	11.3	6.6
Greece	4	0.3	0.3	0.1	0.1	0	89.7	212.9
Hungary	1	0.2	0.1	0	0	0	3.7	1.4
Iceland	0	0	0	3.1	0	3.0	9.0	0
Ireland	68	23.9	12.4	9.1	1.5	9.3	5.3	18.8
Italy	85	11.2	36.4	28.1	4.1	14.2	84.0	41.5
Latvia	0.4	1.3	0	17.8	0	0	0	12.8
Lithuania	2	0.6	11.6	3.7	17.0	7.6	0.7	10.5
Luxemburg	12	3.9	0	3.7	0	0	6.9	6.8
Malta	10	0	4.8	0	2.4	0	0	10.9
Netherlands	3	0.6	149.4	8.6	0.4	0.4	0.9	1.4
Norway	8	0.8	1.6	0.6	2.7	0	0.2	2.3
Poland	1	1.6	2.2	2.9	1.2	3.5	1.7	8.9
Portugal	0.2	0.7	0.1	0	0	0	3.3	16.6
Romania	187	179.5	50.3	2.6	0.2	123.1	283.8	55.3
Slovakia	0.4	0.2	0	0	0	0	1.1	105.2
Slovenia	11	1	0.5	25.3	8.7	0.5	3.4	4.4
Spain	43	9.7	2.7	3.3	1	0.8	3.5	4.8
Sweden	3	3.2	5.5	2.7	2.3	0.3	4.2	4.3
United Kingdom	17	30.4	30.7	2.1	1.4	8.7	4.3	14.5
EU/EEA	60	16.2	20.1	7.1	7.7	9.0	28.3	23.9

n.a. not available.

**Table 2:** Variation in vaccination coverage for the second dose of measles-containing vaccine between 2010 and 2018, 2010 and 2016 and 2016 and 2018.

Country	Variation in Vaccination Coverage for the Second Dose of Measles-Containing Vaccine (%)		
	2010–2018	2010–2016	2016–2018
Czech Republic	−14	−5	−9
Romania	−12	−17	+5
Bulgaria	−9	−8	−1
Estonia	−7	−3	−4
Norway	−4	−6	+2
Netherlands	−3	−1	−2
Croatia	−3	−2	−1
Lithuania	−3	−3	0
Poland	−2	−1	−1
Slovakia	−2	−2	0
Slovenia	−2	−3	+1
Cyprus	0	0	0
Germany	0	0	0
Hungary	0	0	0
Sweden	+1	+1	0
Portugal	+1	0	+1
Latvia	+1	−4	+5
Spain	+2	+3	−1
Belgium	+2	+2	0
Iceland	+3	+3	0
United Kingdom	+4	+5	−1
Denmark	+5	0	+5
Greece	+6	+6	0
Malta	+10	+1	+9
Austria	+15	+20	−5
France	+19	+19	0
Luxemburg	n.a.	n.a.	+4
Italy	n.a.	n.a.	+7
Finland	n.a.	n.a.	+8
Ireland	n.a.	n.a.	n.a.

Countries are ordered on the basis of the VC variations 2010–2018 (from the most negative to the most positive). Countries with mandatory vaccination are indicated in red, those where it is only recommended in green. Data are not complete for 4 countries (n.a.: not available).
